# Photoactivation of olfactory sensory neurons does not affect action potential conduction in individual trigeminal sensory axons innervating the rodent nasal cavity

**DOI:** 10.1371/journal.pone.0211175

**Published:** 2019-08-14

**Authors:** Margot Maurer, Nunzia Papotto, Julika Sertel-Nakajima, Markus Schueler, Roberto De Col, Frank Möhrlen, Karl Messlinger, Stephan Frings, Richard W. Carr

**Affiliations:** 1 Experimental Pain Research, Medical Faculty Mannheim, University Heidelberg, Mannheim, Germany; 2 Centre for Organismal Studies, University Heidelberg, Heidelberg, Germany; 3 Institute for Physiology and Pathophysiology, Friedrich-Alexander University Erlangen-Nuremberg, Erlangen, Germany; 4 Department of Nephrology and Hypertension, Friedrich-Alexander University Erlangen-Nürnberg, Germany; Duke University, UNITED STATES

## Abstract

Olfactory and trigeminal chemosensory systems reside in parallel within the mammalian nose. Psychophysical studies in people indicate that these two systems interact at a perceptual level. Trigeminal sensations of pungency mask odour perception, while olfactory stimuli can influence trigeminal signal processing tasks such as odour localization. While imaging studies indicate overlap in limbic and cortical somatosensory areas activated by nasal trigeminal and olfactory stimuli, there is also potential cross-talk at the level of the olfactory epithelium, the olfactory bulb and trigeminal brainstem. Here we explored the influence of olfactory and trigeminal signaling in the nasal cavity. A forced choice water consumption paradigm was used to ascertain whether trigeminal and olfactory stimuli could influence behaviour in mice. Mice avoided water sources surrounded by both volatile TRPV1 (cyclohexanone) and TRPA1 (allyl isothiocyanate) irritants and the aversion to cyclohexanone was mitigated when combined with a pure odorant (rose fragrance, phenylethyl alcohol, PEA). To determine whether olfactory-trigeminal interactions within the nose could potentially account for this behavioural effect we recorded from single trigeminal sensory axons innervating the nasal respiratory and olfactory epithelium using an isolated in vitro preparation. To circumvent non-specific effects of chemical stimuli, optical stimulation was used to excite olfactory sensory neurons in mice expressing channel-rhodopsin (ChR2) under the olfactory marker protein (OMP) promoter. Photoactivation of olfactory sensory neurons produced no modulation of axonal action potential conduction in individual trigeminal axons. Similarly, no evidence was found for collateral branching of trigeminal axon that might serve as a conduit for cross-talk between the olfactory and respiratory epithelium and olfactory dura mater. Using direct assessment of action potential activity in trigeminal axons we observed neither paracrine nor axon reflex mediated cross-talk between olfactory and trigeminal sensory systems in the rodent nasal cavity. Our current results suggest that olfactory sensory neurons exert minimal influence on trigeminal signals within the nasal cavity.

## Introduction

The sensory innervation of the mammalian nasal cavity by the trigeminal and the olfactory systems endows the nasal epithelium with a broad spectrum of sensory modalities. Trigeminal fibres originating from the ethmoid and nasopalatine nerves [1] detect irritants, temperature changes and mechanical stimuli [2,3], while olfactory receptor neurons respond specifically to odorants and non-specifically to mechanical stimuli [4]. In addition to the extended trigeminal network innervating the nasal respiratory epithelium, the olfactory sensory epithelium also contains trigeminal fibres [5–8] [9]. In co-ordination with the olfactory system, trigeminal chemesthesis contributes to a continual analysis of the composition of the inhaled air for harmful and useful compounds with the trigeminal signaling being implicated in the induction of protective reflexes, pain perception [10] and subsequent behavioural responses.

It is well established that nasal olfactory and trigeminal sensory systems interact with one another on multiple levels, beginning with the fact that most odorants can stimulate trigeminal fibres and that most irritants have an odour [11]. Work on human nasal sensation has led to the concept that chemical stimulation of the nose triggers a multimodal response, which is often described as an integrated afferent signal rather than as two separate streams of trigeminal and olfactory information [11–21]. Most studies have focused on the suppressive effect of trigeminal stimuli that elicit sensations of pungency on the perception of odorants. Similarly, in animal models, the impact of trigeminal activation on olfactory signaling has been examined and release of calcitonin gene-related peptide (CGRP) from trigeminal afferents can suppress excitatory signals in olfactory sensory neurons [5,22]. However, olfactory signaling can also influence signaling in trigeminal sensory neurons [23,24]. For example, the sensory task of spatial localization of odours attributed to activation of trigeminal neurons is enhanced by odorants [25]. Presently, there is no clear understanding of the molecular pathways nor the anatomical sites at which olfactory signaling might affect trigeminal activity. Imaging studies in people indicate overlap of central trigeminal and olfactory processing pathways [26]. Clinical evidence indicates that olfactory stimuli can affect the course of primary headache disorders, in particular in migraine [27–32] for which modulation within the trigeminal brainstem nuclei is implicated [32,33].

Here we systematically explored the effect of olfactory stimulation on trigeminal signaling in the nose. We used anatomical and single fibre electrophysiological techniques to map and characterize the ethmoid nerve innervation of the nasal cavity. To examine directly the influence of olfactory activation on trigeminal signaling we used light to selectively excite olfactory sensory neurons in OMP-ChR2-YFP mice. We were unable to detect any effect on action potential signaling in single trigeminal sensory afferents innervating the nasal cavity during photoactivation of olfactory sensory neurons and conclude that olfactory sensory neurons exert minimal influence on trigeminal signals within the nasal cavity.

## Methods

Animal housing and all experimental procedures were carried out in compliance with the guidelines for the welfare of experimental animals as stipulated by the Federal Republic of Germany. Animal experiments were approved by the Regierungspraesidium Karlsruhe, Germany (approval number 35–9185.81/G-104/16).

### Animals

Transgenic mouse lines were obtained from Thomas Bozza (Northwestern University, Evanston, IL, USA) for OMP-ChR2-YFP mice [34], from David D. McKemy (University of Southern California, San Diego, CA, USA) for TRPM8-eGFP mice [35] and from Rohini Kuner (University Heidelberg, Heidelberg, Germany) for B6.Cg-Gt(ROSA)26Sortm9(CAG-tdTomato)Hze/J Tg(Scn10a-cre)1Rkun [36]. In this article, we will use the abbreviation Scn10ACre-tdTomato to designate this mouse line. Exclusively male C57BL/6N (Charles River Laboratories) mice were used for behavioral experiments. Male Wistar rats were used for both anterograde tracing and functional recordings to assess ethmoid branching (see below).

### Behavioural assessment of volatile chemical stimuli on water consumption

To avoid confounding influences of hormonal changes on olfaction, only male C57BL/6N mice aged from 9–14 weeks were used for behavioral testing [42,43]. The housing facility was kept at a constant temperature of 22 ± 1°C with a 12 h light/dark cycle. To assess the influence of irritant and odorant stimuli, a forced choice paradigm was developed. Two water bottles (ACBT0152, Tecniplast, Hohenpeissenberg, Germany) were positioned inside home cages separated by a distance of 8 cm. Bottles by virtue of their automated manufacture, were identical and and in all respects indistinguishable (height above cage floor, resistance to water flow, hydrostatic pressure determined by starting water volume, nozzle diameter and orientation in cage).

**Fig 1:**
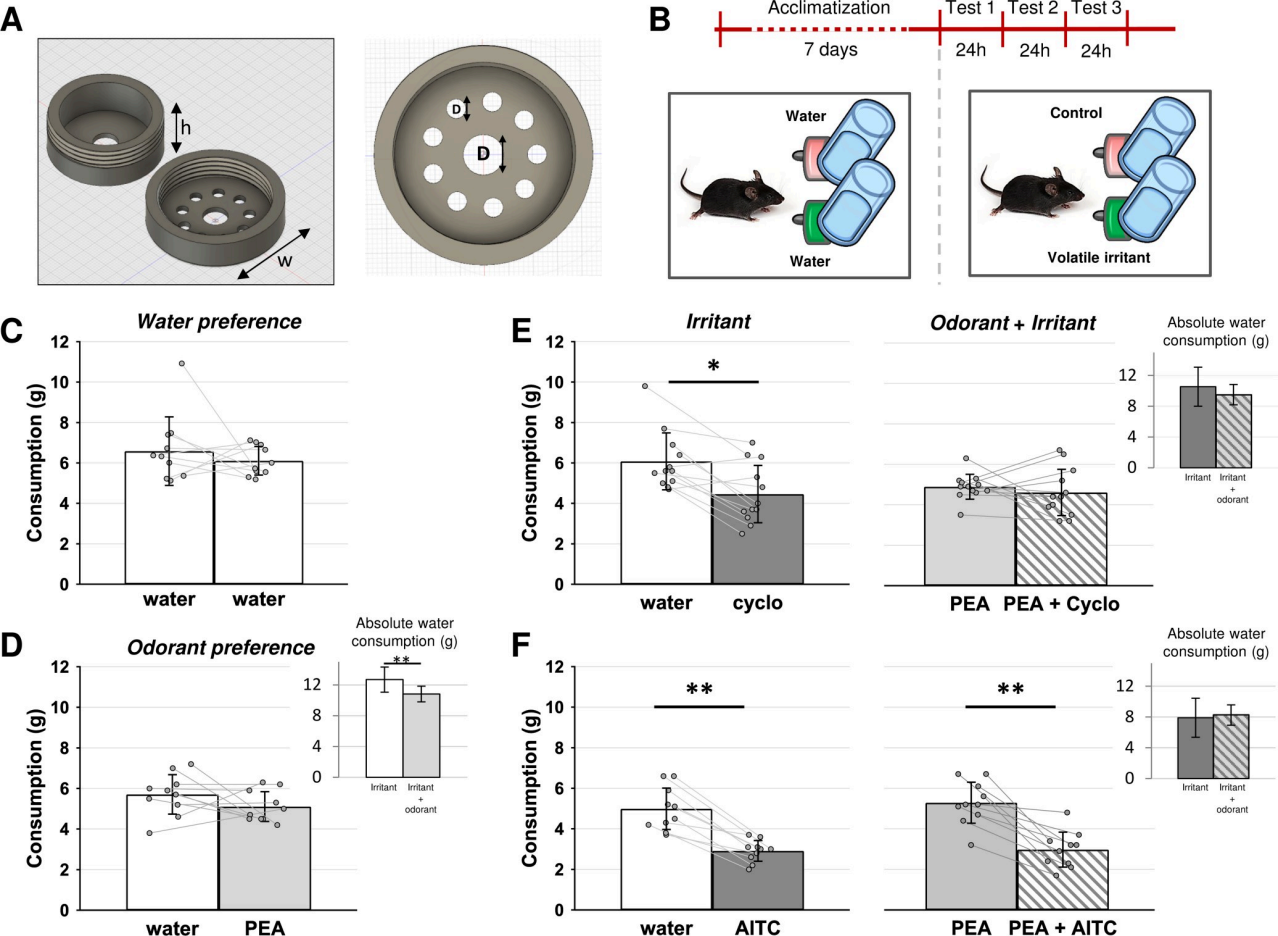
PEA mitigated behavioural aversion to the volatile irritant cyclohexanone in mice. **A:** Depiction of the aluminum annular housing (h = 11 mm; w = 30 mm; D = 8.5 mm and d = 4 mm) that were filled with pieces of felt soaked in irritant and odorant solutions and placd around water sipping tubes in home cages. **B, lower**: Mice had acces to water *ad libitum* from two water sources throughout the experiment. **B, upper**: After seven-days acclimatization to two water sources, housing were filled with an irritant, odorant or vehicle randomized for the side (left/right) over 3 consecutive days. **C**: Water consumption from each of two bottles. In the absence of irritant or odorant there was no bottle preference (paired t-test, n = 10, p = 0.60). **D:** Similarly, there was no drinking preference bottles with pure odorant PEA and vehicle (paired t-test, n = 10, p = 0.26). However, absolute water consumption was diminished by the introduction of PEA (panel D, upper right corner: paired t-test, n = 10, p < 0.01). **E:** Mice showed aversion to the volatile irritant cyclohexanone (panel **E, left**: 2-way ANOVA, factor cyclo, p < 0.05, post hoc Tukey HSD, water vs cyclo, p = 0.01) and this aversion was mitigated with the addition of PEA to both housings (panel **E, right:** 2-way ANOVA, factor cyclo, p < 0.05, post hoc Tukey HSD, PEA vs cyclo, p = 0.94) although absolute water consumption did not change (panel **F, inset upper right corner:** paired t-test, n = 12, p = 0.16). **F:** Mice showed aversion to the volatile irritant AITC (panel **F, left**: 2-way ANOVA, factor AITC, p < 0.01, post hoc Tukey HSD, water vs AITC, p < 0.01) and aversion persisted with the addition of PEA to both housings (panel **F, right:** 2-way ANOVA, factor AITC, p < 0.01, post hoc Tukey HSD, PEA vs PEA + AITC, p < 0.01). Absolute water consumption did not change (panel **F, inset upper right corner:** paired t-test, n = 10, p = 0.40).

Prior to testing, mice were acclimatized for seven consecutive days in individual ventilated polycarbonate cages (32 cm × 16 cm × 30 cm, L × W × D, GM500SU, Tecniplast, Hohenpeissenberg, Germany) with access to food and water from two bottles *ab libitum*. Volatile odorant and irritant stimuli concealed in metal housings were placed on the drinking tubes. Felt rings were soaked in the volatile agents and placed inside the metal housings on day 8. The housings were constructed in house and comprised aluminum annular rings with a perforated screw top lid and a central hole such that the housing could be pushed onto the sipping tubes of water bottles (Fig 1A). This system exposed the immediate vicinity of the drinking tube to a high concentration of volatile compound. Odorant and irritant stimuli remained around the sipping tubes for a period of 24 hours (Fig 1B). Water bottles were washed daily with ethanol and water and refilled with fresh water before being replaced in the cage with a felt washer freshly soaked in odorant or irritant. The amount of water consumed was quantified by establishing the change in weight of each bottle over each consecutive 24-hour period during acclimatization, exposure to volatile agents and in the post-exposure phase.The absolute water consumption was measured daily. Student’s paired t-test was used for paired comparisons of absolute water consumption.

A two-way ANOVA was used to assess interactions between odorants and irritants. The factors were “odorant” and “irritant” corresponding to groupings of “water / irritant” versus “PEA / PEA + irritant” and a grouping of “water / PEA” versus “irritant / irritant + PEA” respectively. F ratio and significance values by factor and their interaction are indicated in the text and Figures.

### Anterograde tracing

**Fig 2:**
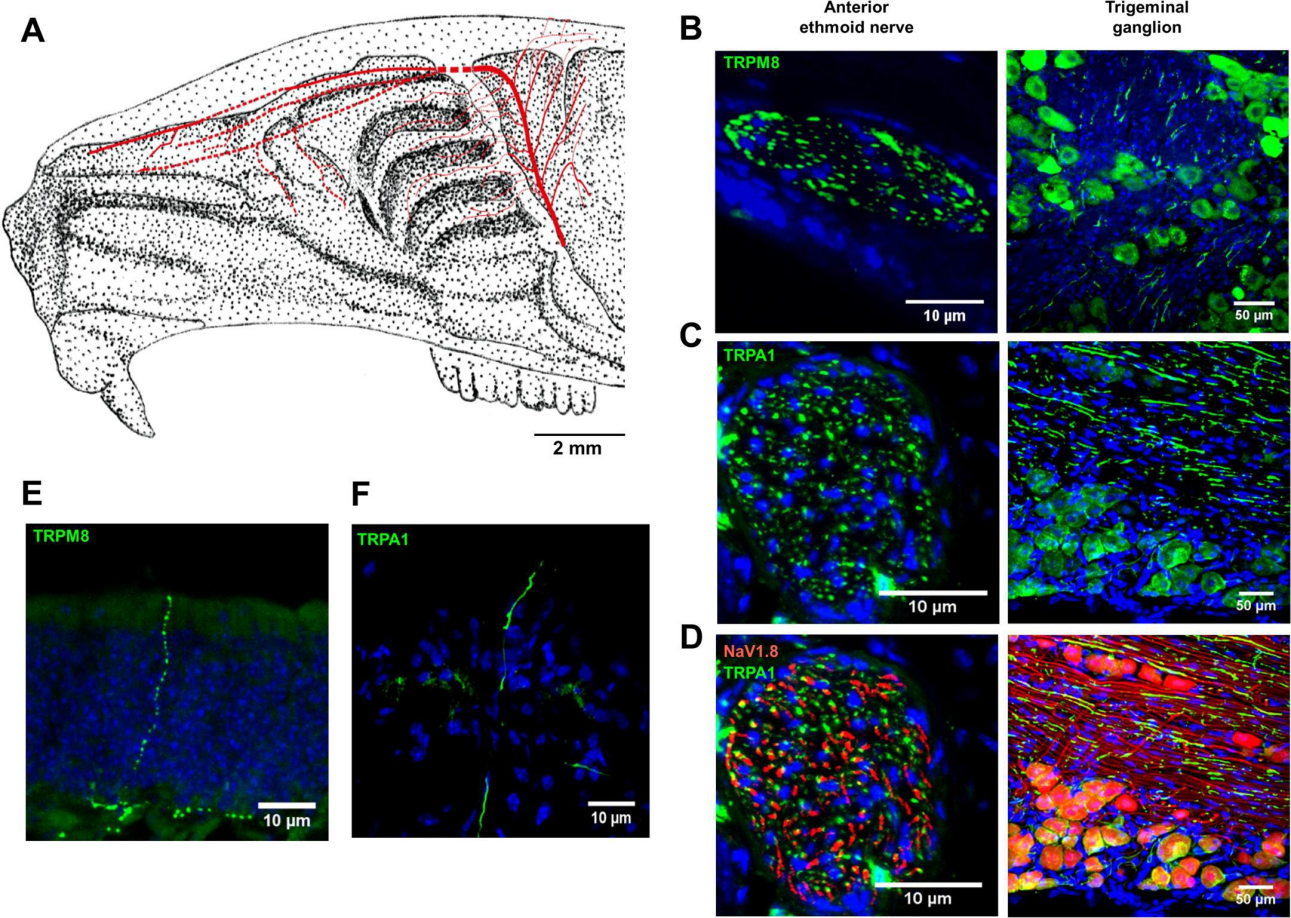
Ethmoid innervation of the rodent nasal cavity. **A**: Spatial distribution of ethmoid nerve revealed by anterograde tracing with the dextran amine DiI (red tracing) overlaid on a sketch of the rat skull in mid-sagittal section (scale bar: 2 mm, background adapted from barrios et al. 2014 [69]).B-D. **B-D:** Representative immunohistological sections of anterior ethmoid nerve (left images, scale bar: 10 μm) and trigeminal ganglion (right images, scale bar: 50 μm) from TRPM8eGFP mice (B, green), TRPA1-immuno-stainings in WT mice (C, green) and NaV1.8::tdTomato mice (D, NaV1.8-red, TRPA1-green). **E, F:** Images from isolated mouse olfactory epithelium showing nerve terminals expressing the TRPM8eGFP reporter (E, green) and TRPA1-immuno-label (F, green). DAPI staining is shown in blue in panels B-F.

The technique of anterograde tracing has been described previously [44]. Briefly, rats were killed by inhaling CO_2_, the head removed, the cranial vault cleared of overlying tissue and subsequently hemisected mid-sagittally. The brain was removed sparing the cranial dura mater and trigeminal ganglion. The ethmoid nerve was dissected free about 2 mm beyond its traverse into the anterior cranial fossa through the ethmoid foramen (Fig 2A). A small crystal of the carbocyanine dye Di-I3 (1,1′Dioctadecyl‐3,3,3′,3′‐Tetramethylindocarbocyanine Perchlorate, D282, Molecular Probes, Eugene, OR, USA) was placed on the distal cut end of the ethmoid nerve and covered with a piece of gelatin sponge (Abgel, Sri Gopal Labs, Mumbai, India) to avoid spreading of the dye. Dye was transported at approximately 2.5 mm/week and to trace the length of anterior ethmoid nerve took around 2–3 months.

### Immunohistochemistry

Mice heads were prepared for immunohistochemistry using the same procedure as that used for electrophysiological experiments (see above). The cranial vault was fixed in 4% paraformaldehyde for 2 hours before preparing the olfactory epithelium according to the deboning protocol previously described Dunston et al. [45]. Trigeminal ganglia and segments of the ethmoid nerve were isolated from half-skulls after removal of the cortices, brainstem and olfactory bulb.

Each sample was successively dehydrated in a 10% sucrose solution (10% (w/v) sucrose, 0.05% (w/v) NaN_3_ in PBS, pH = 7.4) for 2 hours and cryoprotected in 30% sucrose solution (30% (w/v) saccharose, 0.05% (w/v) NaN_3_ in PBS, pH = 7.4) overnight. Olfactory epithelia, ethmoid nerves and trigeminal ganglia were embedded in Optimal Cutting Temperature medium (OCT, Sakura Finetek, CA, USA) and stored at -20°C. 25 μm sections were cut using a cryotome (Thermo Scientific Microm HM 550, Germany) and mounted onto glass slides (Superfrost plus, Thermo Fisher Scientific).

Sections were washed in 0.1M PBS and blocked with 5% Chemiblocker (Millipore, Darmstadt, Germany) comprising 0.5% Triton X-100 and 0.05% NaN_3_ in PBS at pH = 7.4. Primary antibodies (Table 1) were applied overnight in 5% Chemiblocker before incubation with Alexa Fluor 488-labelled goat anti-rabbit (dilution 1:1000, Invitrogen) and 1 μg/ml DAPI (DAPI, dilactate ≥ 98%, Sigma-Aldrich, Germany). Slides were mounted with fluorescence mounting medium (Dako, Agilent, Italy), imaged (Nikon Eclipse 90i/C1, Nikon, Japan) and analyzed (NIS elements, version 4.0; Nikon) using confocal techniques.

**Table 1:** Primary antibodies.

*Antibody*	*Host*	*Supplier*	*Catalogue number*	*Dilution*	*Immunogen*
Anti-GFP	Rabbit (polyclonal)	Abcam (Cambridge, UK)	Ab6556	1:400	Recombinant full length GFP protein
Anti-TRPA1	Rabbit (polyclonal)	Abcam (Cambridge, UK)	ab58844	1:400	Amino acids 1060–1075 of rat TRPA1

### Chemicals

l-menthol, allyl isothiocyanate (AITC), capsaicin, cyclohexanone, phenylethyl alcohol (PEA, 2-phenylethylethanol) and ammonium chloride (NH_4_Cl) were all purchased from Sigma-Aldrich, Munich, Germany. Substances were made up in stock solutions of either DMSO, alcohol or mineral oil (Mineral oil light, 101880364, Sigma-Aldrich, Munich, Germany). Stock solutions were diluted to the required concentration in the perfusing solution on the day of each experiment. Ammonium chloride was made up as a 4.3% (w/v) solution in distilled water and applied as ammonia (NH_3_) vapour.

### Ex vivo nasal cavity preparation

Adult C57BL/6N mice of both sexes and with body weights ranging from 22 to 31 g were anaesthetized with sevoflurane (Abbott, Weisbaden, Germany) in a sealed glass chamber (2 litres volume) and subsequently killed by cervical dislocation. The head and lower jaw were removed, and the cranial vault cleared of overlying skin and muscle. Similar to our previous description of an ex vivo half skull preparation for recordings from meningeal afferents [37], the skull was divided in the sagittal plane with a scalpel. The cortex, brain stem and olfactory bulb were removed along with the nasal septum. Each half skull was embedded in a perspex chamber using agar (8%; Agar-agar, Kobe I, 5210.3, Carl roth GmBH, Karlsruhe, Germany) such that the nasal cavity and the bony cavity of the olfactory bulb formed a contiguous tissue bath. The experimental recording time for each half skull ranged from 2–6 hours.

The half skull bath was perfused continuously at ca. 4 ml.min^-1^ with physiological solution comprising (in mM): hydroxyethyl piperazine ethanesulfonic acid solution (HEPES), 6; NaCl, 118; KCl, 3.2; NaGluconate, 20; D-Glucose 5.6; CaCl_2_, 1.5; MgCl_2_, 1. The pH was adjusted to 7.4 with NaOH. The temperature of the perfusing solution was controlled at 32.0 ± 0.500A0°C with an in-line resistive heating element regulated by feedback from a thermocouple positioned in the bath.

### Recording arrangement

The anterior ethmoid nerve was identified in the anterior cranial fossa along its course within the dura mater from the anterior ethmoid foramen inferiorly to the cribroethmoid foramen rostrally where the nerve enters the nasal cavity [38].

The ethmoid nerve was cut as close to its entering in the cranial vault through the ethmoid foramen as possible and the distal cut end freed of surrounding dura over a length of approximately 4 mm sufficient to attach a glass recording electrode to the cut end by light suction. The glass recording electrode was filled with physiological solution and the tip cut with a sapphire blade to match the diameter of the ethmoid nerve. Signals were recorded over the sealing resistance relative to an Ag/AgCl pellet in the bath using a differential amplifier (NL104A, Digitimer, City, UK). Signals were filtered (low-pass 5 kHz, 80 dB Bessel), digitized (20 kHz, micro 1401, Cambridge Electronic Design, Cambridge, UK) and stored to disk for subsequent analysis.

### Mechanical, electrical, thermal and chemical stimulation

Receptive fields of individual sensory axons in the nasal cavity were established using either a mechanical (von Frey) stimulus (120 μm-diameter, 1.47 mN buckling load, and 130 kPa pressure) or an electrical stimulator without prior mechanical searching. In the case of mechanical searching, a servo driven mechanical stimulator [39] was placed at sites within the area mapped with the von Frey filament. The mechanical stimulator was used to deliver brief sinusoidal (10 ms pulse width) mechanical stimuli at different sites until a single unit response was identified. For electrical stimulation a rayon insulated platinum iridium wire (ISAOHM, Isabellenhütte, Dillenburg, Germany), 20 μm in diameter and exerting a buckling load of ca. 0.4 mN, was placed on the tissue and served as the cathode. A Ag/AgCl pellet (WPI, Sarasota, Florida, USA) positioned in the tissue bath served as the anode for constant current electrical stimuli (1 ms, < 100 μA). Thermal stimuli were delivered by changing the temperature of the solution perfusing the bath. The heating-element bath perfusion circuit had a thermal time constant of approximately 14s. For chemical stimuli, substances were delivered to the solution perfusing the bath, excluding ammonia (NH_3_).

Ammonia (NH_3_) was applied to the nasal cavity in volatile form (and thereby always together with HCl) by dissolving ammonium chloride (NH_4_Cl, 4.3% w/v) in distilled water. Approximately 1ml of NH_4_Cl solution was drawn into a 2ml syringe. To apply the volatile chemical stimulus an approximately 0,2ml volume of air containing the NH_3_ /HCl vapour was expelled from the syringe via a thin tube placed over the nasal cavity. For this series of experiments the half skull was mounted in the recording bath slightly inclined in the sagittal plane, such that the fluid level in the bath could be reduced transiently to expose most of the nasal cavity to air leaving sufficient solution to maintain fluid around the electrophysiological recording pipette attached to the ethmoid nerve within the anterior cranial fossa.

### Determination of axonal conduction velocity

Axonal conduction velocity was calculated by dividing the latency of the action potential response to electrical stimulation by the length of axon between the stimulating and recording sites. The length of nerve between the two sites was estimated visually by reference to a graticule placed in the light path of the microscope’s ocular objective.

### Determination of mechanical threshold

Estimates of mechanical activation threshold were determined for individual axons by determining the likelihood of an action potential response at several discrete stimulus strengths as previously described [39]. Briefly, the probability (number of responses / number of stimuli) of evoking an action potential response across five repeat presentations at each force was determined and the regression of probability on force was fit with a sigmoid function. Mechanical threshold was taken as the inflection point of the fit. Mechanical stimuli were sinusoidal in form and typically of 10 ms duration. The force of mechanical stimulation was taken as the peak maximum of the sinusoidal force profile and force was divided by tip area (200 μM diameter, 0.125 mm^2^) to estimate mechanical stress.

### Evaluation of response to temperature and chemical stimuli

Extracellular recordings of single C-fibres from peripheral nerves are typically performed by teasing the cut end of a nerve manually into progressively smaller filaments. However, the short length of nerve and limited access preclude use of split fibre techniques for the ethmoid nerve in the mouse. We therefore adopted a loose extracellular patch technique to record from the entire ethmoid nerve. In this configuration, signals from multiple units were recorded. To refine this to a single fibre recording a small electrical or mechanical stimulus was delivered to the tissue (olfactory or respiratory epithelium in this case) until we established a time-locked single fibre response from stimuli applied at a single site within the receptive field. Even with a time-locked action potential response, the small amplitude and relative uniform shape of extracellularly recorded C-fibre action potentials make it difficult to discern firing patterns of individual axons. Therefore, to ascertain whether functionally identified single axons responded to thermal or chemical stimuli we used the technique of latency “marking” that relies on an increase in axonal conduction velocity in C-fibres subsequent to each action potential [40]. To utilise this physiological principle, constant frequency electrical stimulation was delivered to the receptive field of an individual axon before and during application of thermal or chemical stimuli. Units were considered to have responded with the generation of action potentials if the latency of response to electrical stimulation increased or if the axon became transiently refractory to electrical stimulation, i.e. no response to electrical stimulation was evident.

### Search for axon collaterals

Previous studies using peripheral dye injections into the olfactory bulb and nasal cavity resulted in double-labelled neurons in the rat trigeminal ganglion and suggested that individual trigeminal axons branch divergently to innervate both the olfactory epithelium and the olfactory bulb [41]. Consequently, we used functional techniques to examine the extent of divergent axonal branching in the ethmoid nerve. Since the olfactory bulb was removed during preparation of the isolated half-skull, axon-reflex signalling was examined for axons innervating the respiratory and olfactory epithelium in the nasal cavity and the dura mater lining the anterior cranial fossa surrounding the olfactory bulb dorso-laterally and termed here “olfactory dura mater”. The first paradigm used the same preparation for recording single afferents from the distal cut end of the ethmoid nerve as described above. Time-locked electrically-evoked action potentials were used to identify receptive fields within either the olfactory and respiratory epithelium or the olfactory dura mater. In addition, the nasal cavity or the olfactory dura mater was stimulated mechanically with a von Frey whisker (buckling load 1.47 nM). Mechanical stimuli were applied over the full spatial extent of the anterior cranial fossa by slowly probing sequential sites. To establish objectively whether action potentials were generated in response to von Frey (1.47 mN) stimulation, the average deviation, i.e. variance, of the recorded signal was determined for consecutive 1 s epochs. Responses were considered to have occurred if the variance exceeded a threshold of 1.25-times the average variance over the preceding 5 minutes.

In a second series of experiments, functional verification of axon collaterals was sought by changing the position of recording to the proximal cut end of the nasociliary branch of the anterior ethmoid nerve at a site immediately distal to its traverse of the cribriform plate through the cribroethmoid foramen. In this configuration, any action potentials recorded in the nasocillary branch of the anterior ethmoid nerve in response to mechanical stimulation of the olfactory dura mater must be travelling anti-dromically via axon reflex between branches of individual axons.

### Data analysis and statistics

Electrical stimulation protocols were tracked online using custom scripts in Spike2 (CED, Cambridge, UK) and analyzed offline (IgorPro, Lake Oswego, OR, USA).

For statistical comparisons between groups Student’s t-test were used. For multiple group comparisons, 2-way ANOVA was used with post-hoc Tukey HSD for pair-wise comparisons within factors. P values less than 0.05 were deemed significant.

## Results

### Behavioural assessment of olfactory and trigeminal chemosensory interaction

The influence of odorant activation of olfactory sensory neurons on trigeminal signal processing was assessed at a behavioural level in mice using a forced-choice water consumption paradigm. Home cages were outfitted with two identical water bottles for daily water consumption. While the water itself was not contaminated, access to the water source could be influenced by the presence of a volatile agent around the sipping tube. Chemical stimuli were applied to a felt ring that was encased in an aluminum housing, annular in form and positioned inside the cage, over the sipping tube, 1 cm from the drinking tip (Fig 1A). Mice were acclimatized to the presence of two water bottles with empty aluminum housings over a seven day period before preference assessment in presence of either an odorant, an irritant or both (Fig 1B). Using commercially available drinking bottles we were able to establish a baseline condition in which the amount of water consumed from the two water sources did not differ (Fig 1C, n = 10, paired t-test, p = 0.60). Addition of the odorant PEA to one bottle reduced the overall water consumption in that cage (cf. Fig 1D, upper right; n = 10, paired t-test, p < 0.01) but did not affect drinking preference within individual cages (Fig 1D, n = 10, ANOVA interaction time*PEA F(1,9) = 2.12, p = 0.15). In contrast to PEA, the presence of irritant compounds resulted in an aversion. For the TRPA1 agonist AITC, there was an aversion in both the presence (Fig 1F, left) and absence (Fig 1F, right) of PEA (n = 10, 2-way ANOVA, factor AITC, F(1,9) = 66.80, df = 39, p < 0.01; factor PEA, F(1,9) = 0.07, df = 39, p = 0.78; factor AITC * PEA, F(1,9) = 0.62, df = 39, p = 0.44). The presence of PEA did not change the absolute consumption of water (Fig 1F, inset upper right corner, n = 10, paired t-test, p = 0.40). For the TRPV1 agonist [46] cyclohexanone there was also an aversion (Fig 1E, n = 12, 2-way ANOVA, factor Cyclohexanone, F(1,11) = 6.99, df = 47, p < 0.05 factor cyclo, post-hoc Tukey HSD water vs cyclohexanone p = 0.01). Similarly, the addition of the odorant did not induce a significant change in the absolute consumption of water (Fig 1E, inset upper right corner, n = 10, paired t-test, p = 0.16). However the aversion to cyclohexanone was mitigated when PEA was added to both water sources (Fig 1E, n = 12, post-hoc Tukey HSD PEA vs PEA + cyclohexanone, p = 0.94). The statistical interaction between PEA and cyclohexanone was not significant (n = 12, ANOVA, interaction PEA*cyclo F(1,11) = 3.44, p = 0.07). Nevertheless, this behavioural effect consolidated the proposal that odorant olfactory stimuli can mitigate aversion to volatile irritants in mice. Accordingly, we designed a protocol to establish whether this cross-modal interaction might occur within the nose.

### Nasal and dural projections of the anterior ethmoid nerve

The dextran amine DiI served as an anterograde tracer [44] to establish the meningeal and intra-nasal innervation arising from the ethmoid nerve in the rat (Fig 2A). To further specify ethmoid axons innervating the nasal cavity, we imaged tissue from wildtype mice after immunostaining for TRPA1 (Fig 2C) and from eGFP reporter lines for TRPM8 (TRPM8^eGFP^, Fig 2B) and a tdTomato reporter for the NaV1.8 voltage-gated sodium channel isoform (Scn10ACre-tdTomato, Fig 2D). In the trigeminal ganglion (Fig 2B and 2C, right panels) and the anterior ethmoid nerve (Fig 2B and 2C, left panels) we observed respectively somata and axons expressing TRPM8 (Fig 2B), TRPA1 (Fig 2C) and NaV1.8 (Fig 2D). Within the olfactory epithelium (Fig 2E and 2F) individual somatosensory nerve terminals positive for TRPM8 (Fig 2E) and TRPA1 (Fig 2F) that traversed the olfactory epithelium from the lamina propria to its apical surface were evident as a single unbranched axon. TRPM8 and TRPA1-positive axons were more often encountered in sections from the posterior reaches of the nose.

### Characterization of the anterior ethmoid innervation of the nasal cavity

**Fig 3:**
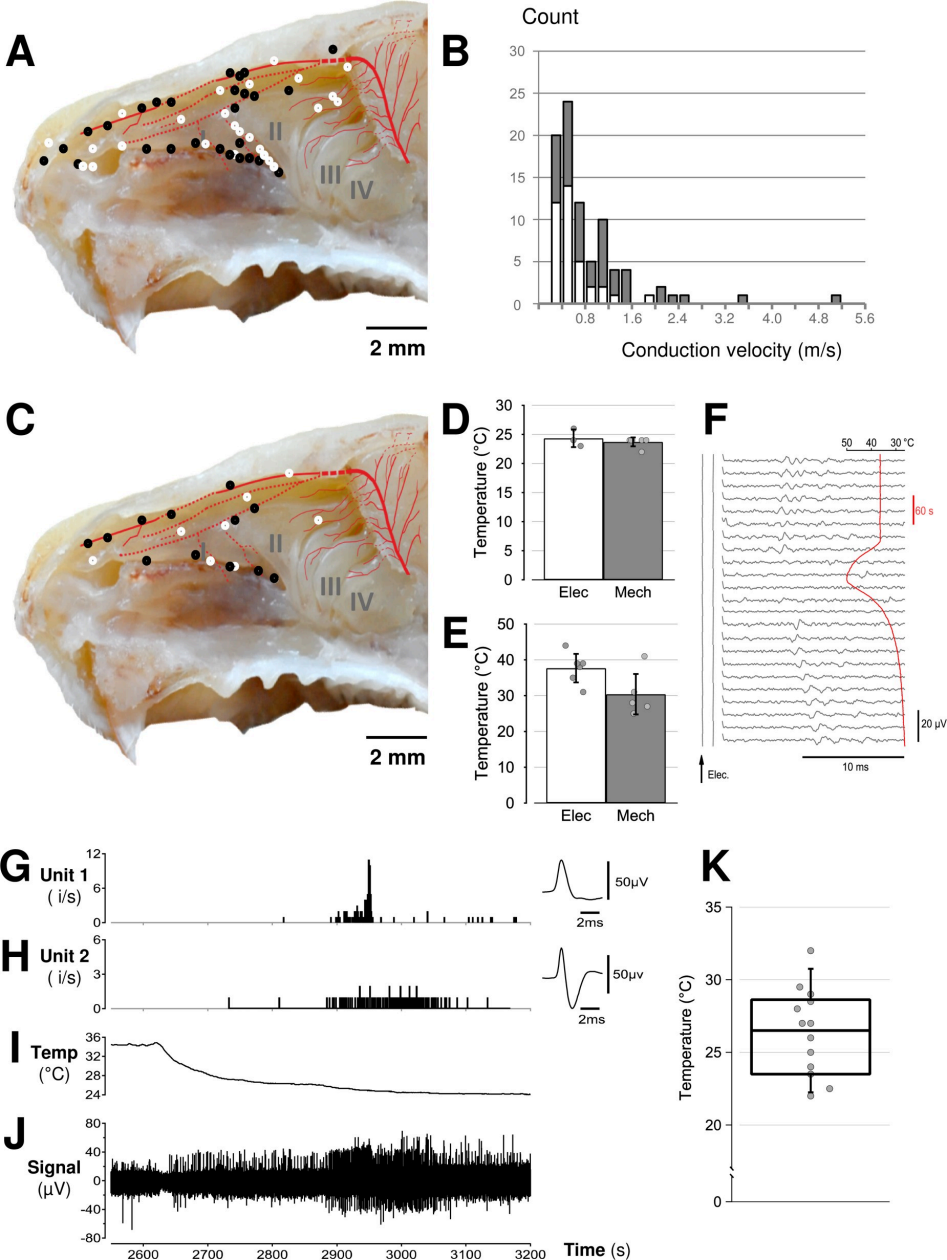
Functional assessment of individual sensory axons in the ethmoid nerve. **A:** Extracellular signals were recorded from axons in the ethmoid nerve with receptive fields in the respiratory and olfactory epithelia identified by electrical (A; white markers) and mechanical (A; black markers) stimuli. (Scale bar: 2 mm;, background adapted from barrios et al. 2014 [69]) **B:** Distribution of axonal velocities for 82 individual trigeminal afferents in the ethmoid nerve. Mechano-sensitive axons are displayed as filled bars (grey; 0.2–5.2 m/s) and electrically-evoked axons are shown as white bars (0.2–2 m/s) **C:** Receptive field sites for thermally sensitive trigeminal axons identified by mechanical (black markers, n = 12) and electrical (white marker, n = 9) search stimuli (scale bar: 2 mm; background image adapted from Barrios et al., 2014). **D&E:** Pooled temperature threshold of trigeminal afferents to heating (D) and cooling (E) and initially identified by mechanical (M, black markers and shading) and electrical (E, white markers and shading) search stimuli. **F:** Falling leaf display of a trigeminal afferent responding to repeat electrical stimulation during a heating and cooling stimulus (red trace). **G-J:** An example of trigeminal afferents as part of a multi-unit recording responding with an increase in firing during cooling (I). Response patterns for two individual units (G&H), identified by spike shape (G&H insets), are shown during cooling. A box and whisker plot representing pooled data for the threshold temperature at which an increase in firing rate was identified in 11 non-tracked units observed in multi-unit recordings (K).

We recorded extracellular action potential signals from the distal cut end of the anterior ethmoid nerve and identified 82 individual axons with mechanical (Fig 3A, black markers) or electrical (Fig 3A, white markers) receptive fields in the nasal cavity. Consistent with structural reports indicating a preponderance of thinly myelinated and unmyelinated axons in the anterior ethmoid nerve [47] we recorded signals from 11 A-delta (≥ 1.5 m/s) axons and 71 C-fibre axons (< 1.5 m/s) [48] with axonal conduction velocities ranging from 0.2 to 5.2 m/s (Fig 3B). Forty-one units were identified using a servo-driven mechano-stimulator. All mechanically-sensitive units excepting one had receptive fields within the respiratory epithelium (Fig 3A, dark circles) and had conduction velocities spanning 0.2 to 5.2 m/s (Fig 3B, dark bar). Absolute mechanical threshold was tested in eight units and ranged from 0.8–8.6 mN. Mechanical receptive fields were punctate, contiguous and comprised areas of approximately 0.1–0.8 mm^2^. Of the 41 mechanically sensitive units, 13 were polymodal. Indeed, 12 individual axons were co-activated by changes in temperature of fluid in the bath (Fig 3C, black markers). Five units responded to heat at 30.4 ± 5.6°C (Fig 3D, dark bar) and 7 units responded to cooling at an average threshold of 23.7 ± 0.7°C (Fig 3E, dark bar).

Using an electrical search stimulus, an additional 41 single units were identified with conduction velocities ranging from 0.2–2 m/s (Fig 3B, white bars). Seventeen of these units were polymodal; 4 responded to mechanical stimuli (0.4 mN) and 9 were temperature sensitive (Fig 3C, white markers). Amongst these axons, 7 units responded to heat (37.9 ± 3.7°C, Fig 3D, white bar) and 3 units responded during cooling (24.3 ± 1.2°C). In addition, responses to cooling were seen in non-tracked trigeminal axons observed as changes in background activity of the recorded signal (see Fig 3J). As an illustrative example, two units, distinguished by their action potential shape are shown in Fig 3G & 3H responding during cooling (Fig 3I). An increase in background activity was observed for 11 recordings during cooling (Fig 3K).

**Fig 4:**
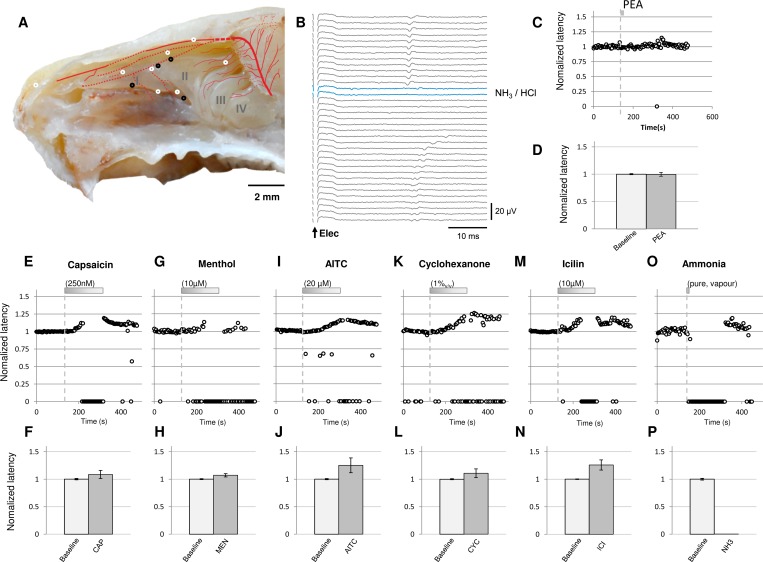
Characterization of responses to chemical stimuli in individual ethmoid afferents. **A:** Receptive field locations within the respiratory and olfactory epithelia for individual trigeminal afferents responding to chemical stimuli and identified using electrical (A; white markers) and mechanical (A, black markers) search stimuli (scale bar: 2 mm;, background adapted from barrios et al. 2014 [69]). **B:** Illustrative example of an individual trigeminal afferent responding at fixed latency to repeat electrical stimulation, shown as a falling leaf display. Application of ammonia (NH_3_, blue trace) activates the axon leaving it refractory to electrical stimulation before it is once again activated and re-establishes a stable response latency to electrical stimulation. **C:** Normalized latency of single afferent response to electrical stimulation across time before, during and after application of phenylethyl alcohol PEA (dashed line) **D:** Pooled data for latency changes in response to pure odorant phenylethyl alcohol (PEA; n = 6, paired t-test, p = 0.77). **E-P:** Representative examples of changes in electrical response latency of individual sensory axons during application of chemical stimuli to the nose (upper panels) and corresponding pooled data for latency changes in response to capsaicin (250 nM; **E&F**, n = 2), (-)-menthol (10μM; **G&H,** n = 1), allyl isothiocyanate (AITC, 20μM; **I&J,** n = 2), cyclohexanone (1%; **K&L**, n = 5), icilin (10μM; **M&N**, n = 2) and ammonia (NH_3_; **O&P**, n = 4).

Sixteen mechanically and electrically-activated units were chemosensitive (Fig 4A) and responded to application of chemical substances in the nasal cavity with a change in latency (Fig 4B). Interestingly, phenylethyl alcohol (PEA) delivered as a vapour did not change response latency (Fig 4C) for any of the 6 units tested (Fig 4D, n = 6, t-test, p = 0.769). However, individual ethmoid axons did respond with a transient increase in latency during application of capsaicin (250 nM; Fig 4E & 4F, n = 2), menthol (10 μM Fig 4G & 4H, n = 1), allyl isothiocyanate (20 μM, Fig 4I & 4J, n = 2), cyclohexanone (1%, Fig 4K & 4L, n = 5), icilin (10 μM, Fig 4M & 4N, n = 2,) and pure ammonia applied as a pressurized air pulse (Fig 4O & 4P, n = 4)

### Influence of olfactory sensory neuron photoactivation on single trigeminal sensory afferents within the nasal cavity

**Fig 5:**
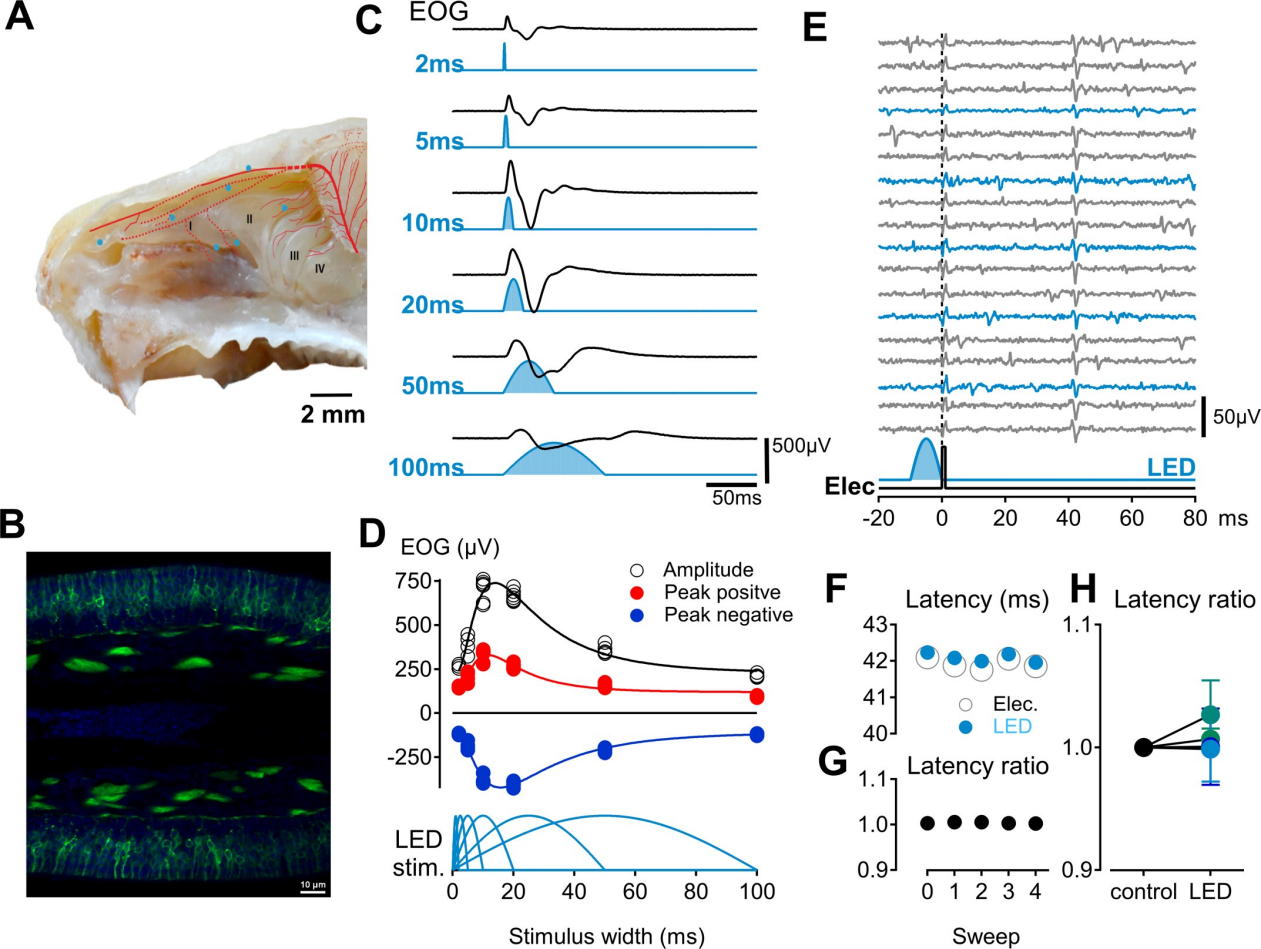
Assessment of functional interactions between olfactory and trigeminal sensory afferents in the nasal cavity. **A**: Electrical receptive field locations for seven trigeminal afferents (A, blue markers) recorded in OMP/ChR2-YFP mice (scale bar: 2 mm, background adapted from barrios et al. 2014 [69]). **B**: Panel B shows the fusion protein ChR2-YFP (green) and nuclear DAPI (blue) staining in the olfactory epithelium in transverse section. **C**: Photoactivation of olfactory sensory neurons was verified by recording extracellular electro-olfactogram (EOG) signals with an electrode positioned on the second turbinate (A, II) in response to sinusoidal light pulses varying in duration from 2-100ms. **D**: The absolute amplitude of the EOG signal was maximal in response to a 14 ms sinusoidal light pulse (D, black trace), while the positive going EOG was maximal for stimuli of 9 ms duration (D; red trace) and the negative-going EOG signal had a maximum amplitude at stimulus widths of 18 ms (D; blue trace). **E**: The response latency of action potentials in single trigeminal axons evoked by electrical stimulation (E, lower black trace) was monitored during electrical stimulation alone every 4s (E, grey traces) and combined with photoactivation (E, lower blue trace) of olfactory sensory neurons every 12s (E, blue traces). **F-G**:The average response latency to electrical stimulation alone (F, open grey markers) was compared to the electrical response latency when applied with light stimulation (F, blue markers) and the ratio of these two latencies determined (G). **H**: Pooled latency ratios for electrically-evoked responses in trigeminal afferents without light stimulation (control) and in combination with photostimulation (LED) are shown for seven fibres.

To dissociate trigeminal and olfactory chemosensory systems we used photostimulation to activate olfactory sensory neurons (OSN) in isolation and synchronously using preparations from OMP/ChR2-YFP mice (Fig 5A & 5B)[49]. To establish the efficacy of photostimulation of OSNs we recorded electro-olfactogram (EOG) signals from the surface of the olfactory epithelium during stimulation of the tissue with sinusoidal pulses of blue light (473 nm; Fig 5C). By varying stimulus pulse width, we found a peak in the EOG amplitude for pulse widths between 10–20 ms (Fig 5D). In addition, prolonged OSN photoactivation elicited an EOG with an initial phasic component and a sustained tonic component (Fig 5C & 5D). We thus used photostimulation pulse widths of 10 ms, corresponding to the most synchronous OSN activation (Fig 5D), and 100 ms, corresponding to the approximate length of a sniffing cycle in the mouse [50]. The effect of OSN photoactivation on single trigeminal afferent signals was determined during a 10 ms light pulse (Fig 5E), corresponding to the peak amplitude of the photo-evoked EOG (Fig 5D) and 100ms, to replicate sustained OSN activation. For seven trigeminal C-fibre axons, the latency of electrically-evoked action potential responses was not altered with repeat applications of a 10–100 ms photoactivation of olfactory sensory neurons (Fig 5E). For comparisons between individual trigeminal afferents we determined the average response to electrical stimulation (grey open markers, Fig 5F) and compared these to the latency of action potentials signals following paired light and electrical stimulation (blue traces, Fig 5E and blue markers, Fig 4F). Taking the ratio of these two latencies (Fig 5G) we saw no effect (paired t-test, n = 7, p = 0.29) of OSN photostimulation on trigeminal axonal conduction (Fig 5H). Consistent with this result, we also observed no change in the electrical response latency of trigeminal afferents during application of the pure odorant phenylethyl alcohol (Fig 4C and 4D).

### Functional assessment of collateral branching in trigeminal sensory axons to the nasal cavity and olfactory dura mater

**Fig 6:**
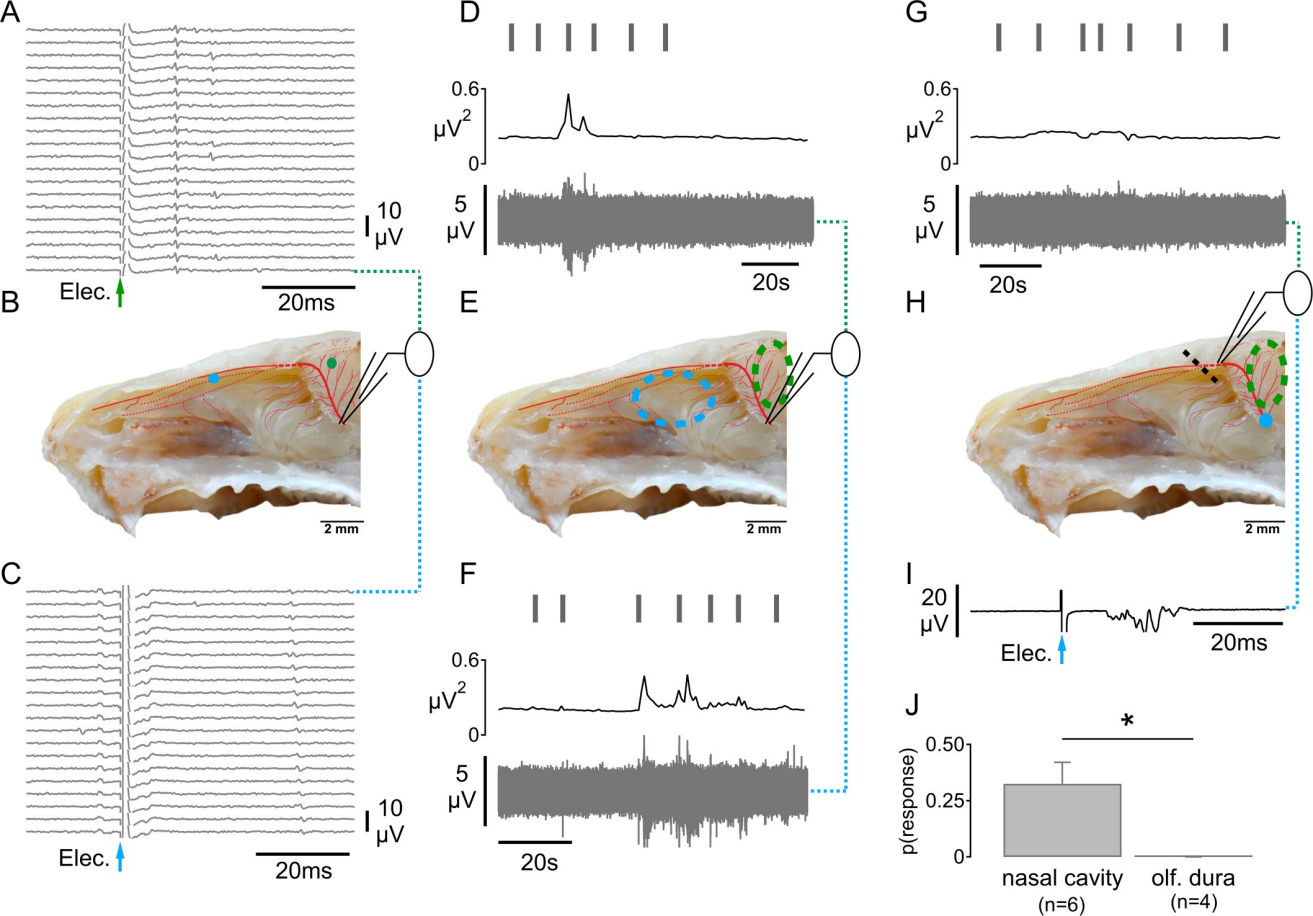
Functional assessment of trigeminal axonal branching to the olfactory epithelium and the olfactory dura mater. **A-C**: Recording from the distal cut end of the ethmoid nerve at its entry into the anterior cranial fossa (B) it was possible to verify electrical (A&C) receptive fields for single trigeminal axons both in the nasal cavity (C) and in the olfactory dura mater (A). **D-F**:Using the same recording configuration, it was possible to verify action potential activity (D & F) in response to mechanical von Frey probing (1.47 mN, D & F, vertical black markers) in the nasal cavity (E, dashed blue circle) and in the olfactory dura mater (E, dashed green circle). Responses to von Frey (1.47 mN) stimulation were assessed by determining the variance of recorded signal over consecutive 1 s bins (D&F, middle traces and see Methods). **G-I**: In the same half-skull preparation as shown in panels D through F, the position of the recording electrode was changed to a more distal site, specifically the proximal cut end of the anterior ethmoid nerve upon its entry into the nasal cavity through the cribroethmoid foramen (H). At this site, it is only possible principally to record anti-dromic activity in the trigeminal axons. We verified this using electrical stimulation to evoke a multi-fibre compound C-fibre action potential response (I) when stimulating at the original site of recording on the ethmoid nerve (H, blue dot). Functional assessment of whether axon reflex signals could propagate from sites in the anterior cranial fossa anti-dromically into the nasal cavity were determined by stimulating with a von Frey filament (G; black vertical markers) at sites within the olfactory dura mater (G, dashed green circle). Mechanical stimulation with a von Frey filament (1.47 mN) did not increase activity as determined by analysis of signal variance (G, centre trace). **J:** Action potential activity was seen in response to random mechanical probing of the nasal cavity but not during von Frey stimulation of the olfactory dura (paired t-test; n = 6, p < 0.01). Scale bar: 2mm, background adapted from barrios et al. 2014 [69].

A functional pathway of communication between the olfactory epithelium and the olfactory bulb has been proposed (Schaefer et al, 2002) in which axon reflex conduction of action potentials allows signaling between sibling branches of individual trigeminal axons in the ethmoid nerve. To examine the possibility of axon reflex conduction between spatially distinct regions of ethmoid nerve innervation, we took advantage of the ability to record from spatially distinct sites along the ethmoid nerve (Fig 6). Owing to removal of the olfactory bulb in our in vitro preparation we opted to examine axon reflex signaling between the nasal cavity and the dura mater lining the anterior cranial fossa, referred to here as the olfactory dura. We began by recording from the distal cut end of the ethmoid nerve immediately distal to its entry point into the anterior cranial fossa through the ethmoid foramen (Fig 6B & 6E). Recording at this site, it was possible to find receptive fields of single C-fibres using an electrical stimulus in either the olfactory dura mater (Fig 6A) or the nasal cavity (Fig 6C). Afferents responses to mechanical stimulation were also observed using a von Frey hair applied manually to sites in the olfactory dura (Fig 6D) and nasal cavity (Fig 6F). This distribution of receptive fields was entirely consistent with the topography revealed by anterograde tracing (Fig 2A) and with previous reports on dural receptive fields overlying the olfactory bulb from single fibres in the nasociliary nerve [51]. Together this established that branches of the ethmoid nerve innervate structures both within the nasal cavity and the cranial meninges lining the anterior cranial fossa.

To test whether this macroscopic divergent branching of the ethmoid nerve comprised a functional signaling pathway, we recorded from the proximal cut end of the ethmoid nerve sectionned immediately distal to its entry point into the nasal cavity (Fig 6H, dashed black line) through the cribroethmoid foramen (Fig 6H). In this configuration, action potential activity recorded from the electrode must be travelling in an anti-dromic fashion, i.e. towards sensory nerve terminals within the nasal cavity. To confirm the possibility of recording action potential activity in this configuration, the stimulus electrode was placed on the parent ethmoid nerve at a site immediately distal to the ethmoid foramen (Fig 6H, blue dot). Constant current stimulation here produced a time-locked compound C-fibre action potential, i.e. multi-unit response (Fig 6I). We then searched the olfactory dura mater (Fig 6H, dashed green outline) with a von Frey probe (Fig 6G, black bars upper). If individual axons branched to both the olfactory meninges and the nasal cavity, action potentials conducted by axon reflex should have been evident at the recording electrode in the nasal cavity in response to mechanical probing of the olfactory dura mater. In six half skull preparations (1 mouse, 5 rat), action potential activity was seen in response to random mechanical probing of the nasal cavity (Fig 6J; paired t-test; p < 0.01, n = 4) but not during von Frey stimulation of the olfactory dura. This suggests that if individual branch do innervate both the olfactory epithelium and olfactory dura mater, it occurs rarely.

## Discussion

This study set out to examine interactions between the dual olfactory and trigeminal chemosensory systems in the nose. Using a forced choice behavioural assay in wild type mice, a combination of odorant and irritant stimuli mitigated the otherwise prominent aversion to irritant stimuli alone. Previous reports suggest that cross-talk between olfactory and trigeminal chemosensory signals might take place within the nose, either through paracrine effects mediated by local release of neurotransmitters [23] or through axon reflex signaling in branched trigeminal afferents [42]. We tested each of these proposals by recording directly from trigeminal axons innervating the nasal cavity. Using optogenetic techniques to activate olfactory sensory neurons exclusively we were unable to discern any modulation of axonal conduction in individual trigeminal afferent axons. We also found no evidence for axon reflex signaling within individual trigeminal axons in the ethmoid nerve that might otherwise form a nexus between the olfactory epithelium and the olfactory bulb. An inability to verify cross-modal interactions between olfactory and trigeminal structures within the nasal cavity suggests that behavioural manifestations of olfactory-trigeminal cross-talk are most likely to occur at more central sites such as trigeminal brainstem nuclei.

Functional recordings from trigeminal axons innervating the nasal cavity have shown that afferent signals are generated in response to a range of stimuli including odorant and irritant chemicals [52–54], mechanical probing in the nostrils [55] and cooling [56]. We verify here, using single fibre techniques and precise spatial stimulus delivery, that trigeminal afferents encode each of these sensory modalities and extend the range of stimuli to include sensitivity to heat. In addition, akin to somatosensory afferents in skin [57] and trigeminal ganglion neuronal somata [58] many trigeminal afferents innervating the nasal cavity are polymodal with heat thresholds around 42°C and mechanical activation thresholds similar to those reported for individual meningeal trigeminal afferents [39,48]. Notably, we saw no evidence of warm fibres but did observe occasional cold-sensitive units as part of the background activity during heating and cooling ramps. The olfactory epithelium receives a lower density of trigeminal afferents than the respiratory epithelium [6] and our functional mapping of trigeminal afferents in the nose was consistent with a lower density in the olfactory epithelium (Fig 3). Trigeminal sensory axons within the mouse respiratory epithelium were also chemosensitive (Fig 3) and this is consistent with previous descriptions of chemosensitive trigeminal neuron somata innervating the nasal cavity selected using viral tracing methods [59].

Psychophysic studies in people indicate that odorants can act as irritants and likewise most irritants have an odour [18]. However, the threshold for chemesthetic trigeminal activation is typically an order of magnitude higher than that for olfactory sensory neurons [54]. While it is well established that odour perception [5], odour detection [21] and odour localization [60] are all affected by concomitant trigeminal activation, there is very little information as to whether olfactory stimulation might affect trigeminal signaling. Recent observation in people using odour localization as an index of trigeminal activation, indicate that pure odors improve localization and this was attributed to an olfactory-trigeminal interaction within the nose [25]. Using an isolated preparation of the mouse nasal cavity we were unable to detect paracrine effects of olfactory sensory neuron activation on trigeminal chemosensory signaling (Fig 4). Although photoactivation of olfactory sensory neurons excludes non-specific actions associated with chemical stimuli, we cannot rule out the possibility that volatile stimuli applied to humans or mice could affect other chemosensory cells in the olfactory epithelium. For example TRPM5-expressing solitary chemosensory cells located in the main olfactory epithelium of rodents [61] are capable of vesicular release of humoural mediators [62] that could act in a paracrine manner on trigeminal nerve terminals. A further confounding factor is the likelihood that the volatile irritant stimuli activate olfactory sensory neurons directly as previously shown for AITC by in vitro electro-olfactogram [5]. Indeed, irritant activation of the olfactory system in our experiments could readily account for the observed behavioral effects of combined irritant and odorant stimuli and this would remain consistent with the lack of effect of olfactory sensory neuronal photoactivation on trigeminal afferents (Fig 5).

In addition to paracrine olfactory-trigeminal interactions within the nasal cavity, Schaefer et al (2002) have suggested that divergent branching of individual trigeminal sensory axons to innervate both the nasal cavity and the olfactory bulb could constitute a pathway subserving trigeminal-olfactory interactions. In this scheme, action potentials generated in the terminals of one branch could affect neurotransmitter release in sister branches by axon reflex. CGRP-positive axons are evident in the olfactory bulb and are of trigeminal origin as evidence by their absence following lesion of the ophthalmic division [63]. Ethmoid peptidergic axons presumably enter the olfactory bulb together with the olfactory tract but may also span the leptomeninges from the olfactory dura mater as observed in rat pups [22,64]. Since our recordings from the ethmoid nerve necessitated removal of the olfactory bulb, we were not able to test the possibility of trigeminal axon branching into the olfactory bulb. Instead we used the olfactory dura mater lining the anterior cranial fossa as a surrogate but failed to observe any evidence of individual axons branching to innervate both olfactory dura mater and the nasal cavity (Fig 6).

Axon reflex mediated release of vasoactive neuropetides is well established in human skin where axon potential spread in cutaneous nerve terminal arbours results in the spreading flare observed around a site of injury [65] and can be blocked by sodium channel blockers [66]. Some functional reports also suggest that proximal axonal branching in individual sensory axons enables innervation of spatially separate tissues [67]. Consistent with this concept, structural studies indicate that trigeminal nerves branch divergently to innervate cranial meninges, cranial bone and extracranial periosteum [64]. Subsequent functional studies using collision techniques confirmed that branches of individual sensory axons in the spinosus nerve gave rise to discrete mechanical and electrical receptive fields in intracranial meningeal structures and in extracranial muscle and fascia [68]. This observation extends the concept that headaches arise from activation of trigeminal afferents innervating the cranial dura mater to one in which the origin of headache includes activation of their axonal projections at extracranial sites. Similarly, irritant chemical stimuli in the nasal cavity could potentially generate action potentials that invade collateral branches in the olfactory dura mater. We adopted functional techniques similar to those used in previous experiments [68] but were not able to confirm the observation made by Schaefer et al. [41] for axons in the ethmoid nerve, at least not for individual axons that might branch to innervate the nasal cavity and the olfactory dura mater (Fig 6). Although it is not possible to assess all axons within the ethmoid nerve, the absence of any retrograde axon reflex action potential activity suggests that if branching in individual axons does occur, and thus forms a functional nexus between these sites, the incidence is likely to be rather low.

Human perception of odours can be modified by the presence of irritants. Similarly, we observed that irritant aversion in mice can be mitigated by co-application of an odorant. Both observations imply an interaction between chemical activation of nasal trigeminal and olfactory pathways at a level sufficient to affect behavior. On the basis of direct recordings from trigeminal sensory axons innervating the nasal cavity, it is not likely that this behavioural effect is causally related to interactions within the nose, implicating trigeminal brainstem nuclei or higher convergent brain areas as sites for sensory cross-talk between trigeminal olfactory chemosensory signaling.

## Supporting information

S1 Table Measures for water consumption preference tests in mice exposed to odorants or irritants. (XLSX)

S2 Table Characterization of individual anterior ethmoidal afferents in the nasal cavity. (XLSX)

S3 Table Latencies of ethmoidal responses in the nasal cavity during optogenetic stimulation of olfactory sensory neurons. (XLSX)

S4 Table Activation of trigeminal receptive fields in the olfactory dura and nasal cavity with a von Frey filament. (XLSX)
